# Association between the IVS4G > T mutation in the *TCF7L2* gene and susceptibility to diabetes in cystic fibrosis patients

**DOI:** 10.1186/1756-0500-5-561

**Published:** 2012-10-10

**Authors:** Daniela Tenório Furgeri, Fernando Augusto de Lima Marson, Antonio Fernando Ribeiro, Carmen Silvia Bertuzzo

**Affiliations:** 1 Department of Medical Genetics, Faculty of Medical Sciences, University of Campinas (UNICAMP), Campinas, SP, Brazil; 2 Department of Pediatrics, Faculty of Medical Sciences, University of Campinas (UNICAMP), Campinas, SP, Brazil; 3 Universidade Estadual de Campinas (UNICAMP), Rua Tessália Vieira de Camargo, 126, Cidade Universitária, Campinas, SP 13081-970, Brazil

**Keywords:** rs12255372, IVS4G > T, TCF7L2, Cystic fibrosis, Diabetes

## Abstract

**Background:**

Clinical complications appear to be a decisive factor for the prognosis of patients. Diabetes is an important complication of cystic fibrosis(CF). In our study we evaluated the association between the IVS4G>T mutation in the *TCF7L2* gene with the presence of diabetes in patients with CF.

**Findings:**

We evaluated 145 patients with CF in relation to the genotype of the IVS4G>T mutation. For this, the PCR method associated with specific enzyme digestion was used. The genotypes G/G, G/T and T/T were observed to have frequencies of 54 (37.2%), 78 (53.8%) and 13 (9%), respectively. There was no association between genotype and the occurrence of diabetes among patients.

**Conclusions:**

In our sample, no association was found between the IVS4G>T mutation in the *TCF7L2* gene and diabetes.

## Findings

### Introduction

Cystic Fibrosis (CF) is a monogenic autosomal recessive disease more common in the Caucasian population. It has the overall prevalence of 1:2500 births [1].

CF occurs due to mutations in the gene *CFTR* (“Cystic Fibrosis Transmembrane Conductance Regulator”) located in the 7q3.1 region, which encodes the CFTR protein. Over 1897 mutations were identified in the *CFTR* gene [2].

The disease is characterized by a higher viscosity of secretions leading to chronic obstructive pulmonary disease, recurrent respiratory tract infections, pancreatic insufficiency, increased concentration of chloride in sweat and male infertility [1].

The clinical features of CF are characterized by clinical heterogeneity; some individuals have early death, while others survive to adulthood [3]. Clinical complications appear to be a decisive factor for the prognosis of patients. Diabetes is an important complication of CF. Diabetes risk increases with age, affecting about 25% of adolescents and 40–50% of adults with CF [4-6]. As CF’s patients are now living longer, diabetes has become the most common systemic complication of CF after lung disease. Diabetes is associated with a significantly worse CF prognosis [6,7], although treatment of diabetes improves nutritional status and pulmonary function [8]. Among CF’s patients, the prevalence of diabetes is approximately tenfold greater (at about one-third the age) than is seen for type 2 diabetes in the general population [6,9].

Grant et al. [10] reported on the association of a common microsatellite (DG10S478) within intron 3 of the transcription factor 7–like 2 gene (*TCF7L2*) with type 2 diabetes in an Icelandic case–control sample and replicated this result in two additional case–control cohorts of white patients. The noncoding single-nucleotide polymorphisms rs12255372 and rs7903146 were in strong linkage disequilibrium with DG10S478 (r2 = 0.95 and r2 = 0.78, respectively) and showed similarly robust associations with type 2 diabetes. The authors recommended that these two single-nucleotide polymorphisms be genotyped in all attempts at replication [11].

In our study we evaluated the association of the rs12255372 polymorphism (IVS4G > T mutation) in *TCF7L2* gene with the presence of diabetes in patients with CF.

### Results

This study was approved by the Ethics Committee of the Faculty of Medical Sciences of University of Campinas - UNICAMP (#528/2008).

The study included 145 patients with CF who are monitored by the Pediatric Pulmonology Outpatient Clinic at UNICAMP.

The genotypes G/G, G/T and T/T were observed with frequencies of 54 (37.2%), 78 (53.8%) and 13 (9%), respectively. The frequency of G allele was 0.64 and the T allele 0.36.

Of the total patients, 72 patients (49.7%) were female and 73 (50.3%) were male, diabetes mellitus was reported in 29 (20.3%) patients and 114 (79.7%) had no comorbidity; meconium ileus was diagnosed in 24 patients (16.6%) and 121 (83.4%) had no comorbidity.

The ethnicity of the patients was respectively, 136 (93.8%), 5 (3.4%) and 4 (2.8%) of Caucasoid, Negroid and mixed race.

For the sample that was screened for the F508del mutation, its frequency was: 37 (25.5%) F508del/F508del, 60 (41.4%) with one F508del allele and 48 (33.1%) patients without the F508del mutation identified.

74 patients (51%) were aged less or equal than 154 months and 71 patients (49%) greater than 154 months.

Association between genotype of the IVS4G > T mutation and the development of diabetes and the presence of meconium ileus (Tables 1, 2) was not found. When the variable presence of the F508del mutation was introduced, an association was still not found in our sample (Tables 3, 4).

**Table 1:** **Association IVS4G > T mutation with clinical variables in cystic fibrosis patients followed at the Pediatric Clinic at UNICAMP distribution without the mutations in the *CFTR *gene**

**Sex**	**Male**	**Female**	**Chi-square**	**p-value**
G/G	28 (51.9%)	26 (48.1%)	0.144	0.93
G/T	39 (50%)	39 (50%)		
T/T	7 (53.8%)	6 (46.2%)		
Diabetes	No	Yes		
G/G	39 (72.2%)	15 (27.8%)	3.018	0.221
G/T	64 (84.2%)	12 (15.8%)		
T/T	11 (84.6%)	2 (15.4%)		
Meconium ileus	No	Yes		
G/G	47 (87%)	7 (13%)	1.014	0.602
G/T	64 (82.1%)	14 (17.0%)		
T/T	10 (76.9%)	3 (23.1%)		
Age	≤ 154 months	> 154 months		
G/G	28 (51.9%)	26 (48.1%)	2.141	0.343
G/T	37 (47.4%)	41 (52.6%)		
T/T	9 (69.2%)	4 (30.8%)		

**Table 2:** **Association IVS4G > T mutation in *TCF7L2 *gene after genotypic groupings with clinical variables in cystic fibrosis patients followed at the Pediatric Clinic at UNICAMP distribution without the mutations in the *CFTR *gene**

**Sex**	**Male**	**Female**	**Chi-square**	**p-value**
G/G	26 (48.1%)	28 (51.9%)	0.078	0.864
G/T and T/T	46 (50.5%)	45 (49.5%)		
Diabetes	No	Yes		
G/G	39 (72.2%)	15 (27.8%)	3.017	0.091
G/T and T/T	75 (84.3%)	14 (15.7%)		
Meconium ileus	No	Yes		
G/G	47 (87%)	7 (13%)	0.802	0.489
G/T and T/T	74 (83.4%)	7 (18.7%)		
Age	≤ 154 months	> 154 months		
G/G	28 (51.9%)	26 (48.1%)	0.23	1
G/T and T/T	46 (50.5%)	45 (49.5%)		

**Table 3:** **Association IVS4G > T mutation in *TCF7L2 *gene, distributed by genotype for the mutation in the *CFTR *gene delF508 with clinical variables in cystic fibrosis patients followed at the Pediatric Clinic at UNICAMP with the distribution by mutations in the *CFTR *gene**

**F508del**	**Sex**	**Male**	**Female**	**Chi-square**	**p-value**
-/-	G/G	8 (53.3%)	7 (46.7%)	0.756	0.685
	G/T	12 (42.9%)	16 (57.1%)		
	T/T	3 (60%)	2 (40%)		
F508del/-	G/G	13 (48.1%)	14 (51.9%)	0.932	0.627
	G/T	17 (60.7%)	11 (39.3%)		
	T/T	3 (60%)	2 (40%)		
F508del/F508del	G/G	5 (41.7%)	7 (58.3%)	0.176	0.916
	G/T	1 (33.3%)	2 (66.7%)		
	T/T	16 (43.2%)	21 (56.8%)		
F508del	Diabetes	No	Yes		
-/-	G/G	12 (80%)	3 (20%)	1.356	0.508
	G/T	21 (77.8%)	6 (22.2%)		
	T/T	5 (100%)	-		
F508del/-	G/G	19 (70.4%)	8 (29.6%)	2.858	0.24
	G/T	24 (88.9%)	3 (11.1%)		
	T/T	4 (80%)	1 (20%)		
F508del/F508del	G/G	8 (66.7%)	4 (33.3%)	2.042	0.36
	G/T	18 (86.4%)	3 (13.6%)		
	T/T	29 (78.4%)	8 (21.6%)		
F508del	meconium ileus	No	Yes		
-/-	G/G	15 (100%)	-	2.286	0.319
	G/T	25 (89.35)	3 (10.7%)		
	T/T	5 (100%)	-		
F508del/-	G/G	22 (81.5%)	5 (18.5%)	0.073	0.964
	G/T	22 (78.6%)	6 (21.4%)		
	T/T	4 (80%)	1 (20%)		
F508del/F508del	G/G	10 (83.3%)	2 (16.7%)	3.335	0.189
	G/T	17 (77.3%)	5 (22.75)		
	T/T	1 (33.3%)	2 (66.7%)		
F508del	Age	≤ 154 months	> 154 months		
-/-	G/G	4 (26.7%)	11 (73.3%)	0.813	0.666
	G/T	6 (21.4%)	22 (78.6%)		
	T/T	2 (40%)	3 (60%)		
F508del/-	G/G	15 (55.6%)	12 (44.45)	2.641	0.267
	G/T	12 (42.9%)	16 (57.1%)		
	T/T	4 (80%)	1 (20%)		
F508del/F508del	G/G	9 (75%)	3 (25%)	1.37	0.504
	G/T	19 (86.4%)	3 (13.6)		
	T/T	3 (100%)	-		

(-) absence of F508del mutation.

**Table 4:** **Association IVS4G > T mutation in *TCF7L2 *gene after genotypic groupings, distributed by genotype for the mutation in the *CFTR *gene delF508 with clinical variables in cystic fibrosis patients followed at the Pediatric Clinic at UNICAMP with the distribution by mutations in the *CFTR *gene**

**F508del**	**Sex**	**Male**	**Female**	**Chi-square**	**p-value**
-/-	G/G	8 (53.3%)	7 (46.7%)	0.257	0.613
	G/T and T/T	15 (45.5%)	18 (54.5%)		
F508del/-	G/G	13 (48.1%)	14 (51.9%)	0.931	0.335
	G/T and T/T	20 (60.6%)	13 (39.4%)		
F508del/F508del	G/G	5 (41.7%)	7 (58.3%)	0.018	0.893
	G/T and T/T	11 (44%)	14 (54%)		
F508del	Diabetes	No	Yes		
-/-	G/G	12 (80%)	3 (20%)	0.01	0.919
	G/T and T/T	26 (81.3%)	6 (18.8%)		
F508del/-	G/G	19 (70.4%)	8 (29.6%)	2.652	0.103
	G/T and T/T	28 (87.5%)	4 (12.5%)		
F508del/F508del	G/G	8 (66.7%)	4 (33.3%)	1.437	0.231
	G/T and T/T	21 (84%)	4 (16%)		
F508del	Meconium ileus	No	Yes		
-/-	G/G	15 (100%)	-	1.455	0.228
	G/T and T/T	30 (90.9%)	3 (9.1%)		
F508del/-	G/G	22 (81.5%)	5 (18.5%)	0.067	0.795
	G/T and T/T	26 (78.8%)	7 (21.2%)		
F508del/F508del	G/G	10 (83.3%)	2 (16.7%)	0.566	0.452
	G/T and T/T	18 (72%)	7 (28%)		
F508del	Age	≤ 154 months	> 154 months		
-/-	G/G	4 (26.7%)	11 (73.3%)	0.032	0.857
	G/T and T/T	8 (24.2%)	25 (75.8%)		
F508del/-	G/G	15 (55.6%)	12 (44.4%)	0.297	0.586
	G/T and T/T	16 (48.5%)	17 (51.5%)		
F508del/F508del	G/G	9 (75%)	3 (25%)	1.009	0.315
	G/T and T/T	22 (88%)	3 (12%)		

(-) absence of F508del mutation.

### Discussion

Florez et al. (2006) [11], examined whether the two most strongly associated variants (rs12255372 and rs7903146) predict the progression to diabetes in 3.548 persons with impaired glucose tolerance who were enrolled in the Diabetes Prevention Program, in which lifestyle intervention or treatment with metformin was compared with placebo. The data found showed that the risk alleles in rs7903146 and rs12255372 predict the risk of diabetes prospectively, beyond that conferred by the clinical risk factors.

In 2009, Blackman et al., [6] had already tested whether a family history of type 2 diabetes affected diabetes risk in CF patients in 539 families in the CF Twin and Sibling family-based study. 998 patients were evaluated from the family-based study and 802 unrelated CF patients in an independent case–control study. Family history of type 2 diabetes was shown to increase the risk of diabetes in CF (OR 3.1; p = 0.0009). A variant in *TCF7L2* associated with type 2 diabetes (the T allele at rs7903146) was associated with diabetes in CF in the family study (p = 0.004) and in the case–control study (p = 0.02; combined p = 0.0002). In the family-based study, variation in *TCF7L2* gene increased the risk of diabetes about threefold (HR 1.75 per allele, 95% CI 1.3–2.4; p = 0.0006), and decreased the mean age at diabetes diagnosis by 7 years. In CF patients not treated with systemic glucocorticoids, the effect of *TCF7L2* was even greater (HR 2.9 per allele, 95% CI 1.7–4.9, p = 0.00011). A genetic variant conferring risk for type 2 diabetes in the general population is a modifier of risk for diabetes in CF.

Given these results, it became interesting to evaluate the other polymorphism, rs12255372 that is also related to a risk of diabetes among CF’s patients. Our analysis, however, found no such correlation.

The absence of a relationship may be due to the sample size, but since this correlation is of great importance, it should have appeared even in a small sample. In any case, with an analysis of a larger number of patients it is possible that a relationship between the studied polymorphism and CF risk can still be evidenced.

### Patients and methods

Patients were included in the study from the Pediatric Clinic at the Faculty of Medical Sciences of UNICAMP. The sample size calculation was performed by G-POWER program version 3.1 (using 0.05 alpha, 0.8 beta and W size effect of 0.3). To have the statically power to all analyses using chi square test we need a population size of 143 patients. All patients were confirmed as having CF through two positive sodium and chloride sweat tests (value greater than 60 mmol/L) and by analysis of differential membrane epithelium of the intestine by the dosage of active CFTR through the Ussing chamber. The identification of mutations in the *CFTR* gene was performed in the laboratory of Molecular Genetics, FCM/UNICAMP, which has the routine analysis tests for major mutations found in the population of Brazil, which are: F508del, G542X, R1162X, N1303K, G551D and N1303K. Of the patients initially included in the survey, only those without clinical data for statistical analysis and/or those who did not sign the informed consent were excluded.

The DNA was obtained by the extraction technique of phenol chloroform from 8 mL of venous blood. The concentration of DNA used for analysis was 50 ng/mL. Genotyping was performed using the PCR technique associated with specific enzyme digestion.

The PCR reaction had a 25μL final volume with 100ng of DNA, 1μM of each primers, 200mM deoxynucleotide triphosphates, 1.3mM MgCl2, 50mM KCl, 10mM Tris - HCl (pH 8.4 at 25°C), 0.1% Triton X-100 and 0.35 U Taq DNA polymerase. A pair of primers were designed and optimized to amplify the region of the mutation IVS4G > T, resulting in amplification of 337 bp (S, 5′-CTGGAAACTAAGGCGTGAGG-3′, AS, 5′-TTGTTGAGCTTTACTGAGAT-3′). The procedure for the thermal cycling consisted of initial denaturation at 94°C for 7 min, subsequent denaturation at 94°C for 30′, annealing at 56°C for 45′, and extension at 72°C for 2 min, repeated for 35 cycles, followed by a final extension at 72°C for 7 min.

The product from the PCR had a size of 337 bp and after digestion by the restriction enzyme Tsp509I, fragments of 143 bp + 99 + 95 - G/G genotype, fragments of 126 + 17 + 99 + 95 bp - T/T genotype and fragments of 143 + 126 + 99 + 95 + 17 bp - genotype G/T were found.

Statistical analysis was performed using the Statistical Package for Social Sciences (SPSS) v.17.0 and Open Epi v.5.0 program. Data was compared by different tests according to data distribution. Initially, data was compared the chi square and Fisher exact test. For all the analyses we adopted the value of p = 0.05.

## Competing interests

The authors declare that they have no competing interests.

## Authors’ contributions

DTF carried out the molecular genetic analysis, participated in the sequence alignment and drafted the manuscript. FALM participated in the design of the study and performed the statistical analysis. AFR and CSB conceived of the study, and participated in its design and coordination and helped to draft the manuscript. All authors read and approved the final manuscript.
